# Ubiquitin-specific protease TRE17/USP6 promotes tumor cell invasion through the regulation of glycoprotein CD147 intracellular trafficking

**DOI:** 10.1016/j.jbc.2022.102335

**Published:** 2022-08-02

**Authors:** Yukino Ogura, Norihiko Ohbayashi, Yasunori Kanaho, Atsushi Kawaguchi, Yuji Funakoshi

**Affiliations:** 1 Department of Physiological Chemistry, Faculty of Medicine and Graduate School of Comprehensive Human Sciences, University of Tsukuba, Tsukuba, Japan; 2 Department of Infection Biology, Faculty of Medicine and Graduate School of Comprehensive Human Sciences, University of Tsukuba, Tsukuba, Japan

**Keywords:** intracellular trafficking, deubiquitylation, ubiquitin-specific protease (USP), cell invasion, cancer biology, ABC, aneurysmal bone cyst, CIE, clathrin-independent endocytosis, DUB, deubiquitylating enzyme, ECM, extracellular matrix, IFN, interferon, MARCH, membrane-associated RING-CH, MMP, matrix metalloproteinase, NF, nodular fasciitis

## Abstract

Disordered expression and distribution of plasma membrane proteins at the cell surface leads to diverse malignant phenotypes in tumors, including cell invasion. The ubiquitin-specific protease TRE17/USP6, an oncogene identified in Ewing sarcoma, is highly expressed in several cancers and locally aggressive tumor-like lesions. We have previously demonstrated that TRE17 regulates the trafficking of plasma membrane proteins that enter cells *via* clathrin-independent endocytosis (CIE); TRE17 prevents CIE cargo proteins from being targeted to lysosomes for degradation by deubiquitylating them. However, functional insights into the effects of TRE17-mediated CIE cargo trafficking on cell invasion remain unknown. Here, we show that increased expression of TRE17 enhances invasiveness of the human sarcoma cell line HT-1080 by elevating the cell surface levels of the membrane glycoprotein CD147, which plays a central role in tumor progression. We demonstrate overexpression of TRE17 decreases ubiquitylated CD147, which is accompanied by suppression of CD147 transport to lysosomes, resulting in the stabilization and increase of cell surface-localized CD147. On the other hand, we show knockdown of TRE17 decreases cell surface CD147, which is coupled with reduced production of matrix metalloproteinases, the enzymes responsible for extracellular matrix degradation. Furthermore, we demonstrate that inhibition of CD147 by a specific inhibitor alleviated the TRE17-promoted tumor cell invasion. We therefore propose a model for the pathogenesis of TRE17-driven tumors in which TRE17 increases CD147 at the cell surface by preventing its lysosomal degradation, which in turn enhances matrix metalloproteinase synthesis and matrix degradation, thereby promoting tumor cell invasion.

Dysregulation of plasma membrane proteins leads to disturbance of signal transduction, which causes diverse disorders, including neoplastic diseases (1). Cell surface levels and distribution of membrane proteins are controlled by protein turnover as well as the biosynthetic pathway. Proteins at the cell surface are internalized through clathrin-mediated endocytosis or clathrin-independent endocytosis (CIE) (2). Subsequently, they converge into Rab5A-positive endosomes, wherein a series of sorting events determines the fate of the internalized proteins: they are transported to lysosomes for degradation or recycled back to the plasma membrane (2). Therefore, endocytosis and following intracellular trafficking are crucial processes for the control of the amount and localization of proteins on the cell surface.

Plasma membrane proteins containing the clathrin adaptor-binding motif are internalized *via* clathrin-mediated endocytosis, while others lacking such a specific motif enter cells through CIE. We and others have demonstrated that the reversible ubiquitylation-deubiquitylation cycle of CIE cargo proteins decides itineraries of these proteins: CIE cargo proteins ubiquitylated by the E3 ligase membrane-associated RING-CH (MARCH) family are routed to lysosomes for degradation, whereas the ubiquitin-specific protease TRE17 (also known as USP6) counteracts this process by removing the ubiquitin chains and promotes recycling of the cargo proteins, thereby increasing their abundance on the cell surface (3, 4). Thus, MARCH and TRE17 reciprocally regulate the cell surface level of membrane proteins and their downstream signaling.

*TRE17* was originally identified as an oncogene and its expression is markedly elevated in a wide variety of neoplastic lesions (5, 6, 7). Chromosomal translocation of the *TRE17* locus leading to overexpression of its wildtype protein is associated with various bone and soft tissue neoplastic lesions, especially in aneurysmal bone cyst (ABC) and nodular fasciitis (NF), in which *TRE17* is translocated nearly 70% and more than 90% of cases, respectively (5, 8, 9, 10). ABC and NF are locally aggressive tumor-like lesions characterized by invasive destruction of surrounding tissues accompanied by the expansion of the lesions (6, 11). Overexpression of TRE17 is also observed in several types of malignant cancers (12, 13) and promotes cancer cell invasion and metastasis in colon cancer, and thus, high expression of TRE17 predicts poor survival in patients with colon cancer (14). Increased invasiveness of neoplasms overexpressing TRE17 is likely due to induction of matrix metalloproteinases (MMPs), the enzymes which degrade extracellular matrix (ECM) (15, 16). Recent studies have revealed several pathways, including NF-κB, Wnt, Jak1-STAT3, and c-Jun, through which TRE17 promotes tumorigenesis (15, 16, 17, 18, 19). However, exact CIE cargo proteins regulated by TRE17 and the downstream signaling pathways responsible for the promotion of cell invasion remain unknown.

Tumor cell invasion requires the destruction of mesenchymal collagen or the endothelial basement membrane, which allows the cells to invade neighboring tissues (20). In this process, signal transduction mediated by plasma membrane proteins and ECM degradation by MMPs are important. CD147/Basigin, also known as an extracellular matrix metalloproteinase inducer (EMMPRIN), is a transmembrane glycoprotein of the immunoglobulin superfamily, which induces MMP production in response to extracellular stimuli (21, 22, 23, 24). CD147 is overexpressed in most types of cancers and enhances tumor growth and metastasis *via* its capacity to induce expression of MMPs and to modify the tumor microenvironment (24, 25). It has been well documented that the quantity of CD147 at the cell surface determines the intensity of the CD147-MMPs signaling and is correlated with malignancies of the tumors (26). Recent studies have demonstrated that the facilitation of endocytic recycling of CD147, mediated by the small GTPases Arf6, Rab5 and Rab22, increases surface CD147 in hepatocellular carcinoma and lung cancer, thereby enhancing their MMP production and malignant phenotypes (27, 28, 29). Notably, it has been reported that CD147 is internalized through CIE and recycled through Rab22A-dependent tubular endosomes that are a hallmark of the CIE cargo trafficking pathway (30, 31), indicating that clathrin-independent endocytic recycling plays a crucial role in CD147-dependent tumor cell malignancies.

Our previous finding that TRE17 promotes recycling of CIE cargo proteins (4) raises a question of which CIE cargo protein(s) is responsible for TRE17-mediated tumor cell invasion. In this study, we investigate the role of TRE17 in the intracellular trafficking of CD147 that would contribute to tumor cell invasion. We find that TRE17 prevents CD147 from routing to lysosomal degradation by deubiquitylating this protein, resulting in the increase of cell surface CD147. We also show that pharmacological inhibition of CD147 suppresses the TRE17-promoted tumor cell invasion. This work identifies the first deubiquitylating enzyme (DUB) for CD147 and proposes a novel mechanism for invasiveness of the neoplasms driven by TRE17 overexpression.

## Results

### TRE17 promotes invasion of sarcoma cells in a DUB activity–dependent manner

It has been reported that TRE17 is highly expressed predominantly in neoplasms of mesenchymal origin, including ABC, NF, osteoblastoma, myofibroma, and Ewing sarcoma (5, 8, 10, 32). We therefore employed the human sarcoma cell line HT-1080 cells, which is derived from a mesenchymal tumor with high invasive potential, to investigate the role of TRE17 in cell invasion.

**Figure 1:**
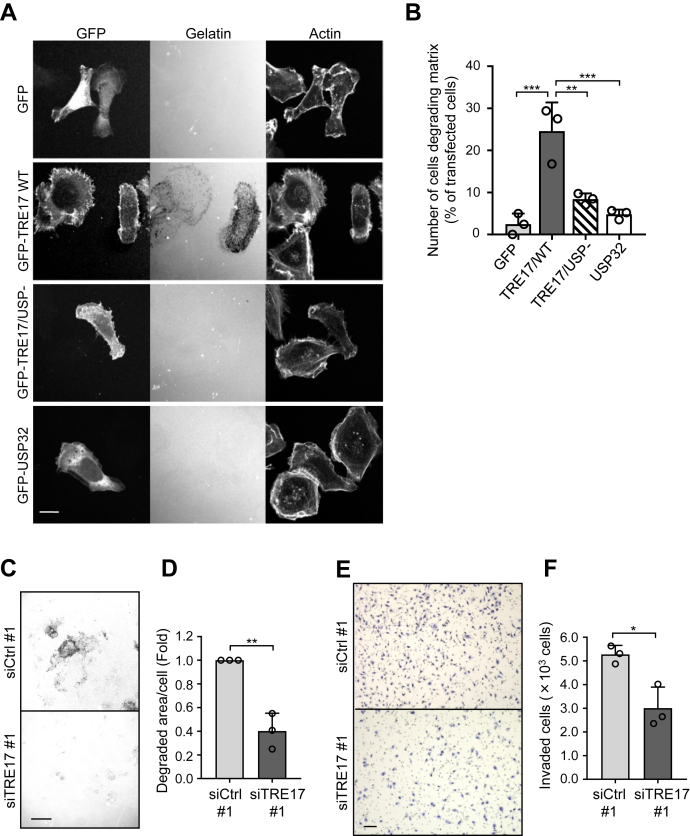
**TRE17 promotes cell invasion in a DUB activity-dependent manner.***A*, HT-1080 cells transfected with GFP, GFP-TRE17 WT, GFP-TRE17/USP-, or GFP-USP32 were seeded onto TRITC-gelatin-coated coverslips and incubated for 2 h. The cells were fixed, stained with Alexa Fluor 633 phalloidin. Scale bar, 10 μm. *B*, quantification of *A*. Transfected cells were randomly selected (80–100 cells), and the number of cells degrading gelatin was counted and plotted as the percentage of transfected cells. *C*, HT-1080 cells transfected with control (siCtrl #1) or TRE17 (siTRE17 #1) siRNA were subjected to the gelatin degradation assay as described in *A*, except for the incubation time (4 h). Scale bar, 100 μm. *D*, to quantify gelatin degradation in *C*, ten fields were randomly selected from the image of TRITC-gelatin. The gelatin-degraded area was normalized with the total cell number and presented as the fold change compared to the control. *E*, HT-1080 cells transfected with siCtrl #1 or siTRE17 #1 were subjected to the cell invasion assay using Transwell chambers. Cells were seeded onto the upper chamber coated with Matrigel and incubated for 20 h. Cells invaded into Matrigel and went through the Transwell filter were stained with hematoxylin. Scale bar, 100 μm. *F*, the number of invaded cells was counted in randomly selected ten fields from *E* and plotted. All the graphs shown are mean ± SD from three independent experiments. ∗*p* < 0.05, ∗∗*p* < 0.005, ∗∗∗*p* < 0.001, one-way ANOVA with post hoc Tukey’s multiple comparison test for *B* and Student’s *t* test for *D* and *F*.

Although TRE17 has been reported to promote invasion of HeLa cells and colon cancer cells (14, 16), it is unclear whether TRE17 is involved in the invasion of tumor cells originated from mesenchymal tissues. Therefore, we assessed the effect of TRE17 on the invasion of HT-1080 cells by observing the degradation of fluorescently labeled gelatin. This assay represents a model of ECM degradation by invasive cells, the critical step of cell invasion. Because of the high motility rate of HT-1080 cells, the cells were cultured for a short period of time (2 h) to prevent each cell from moving out of the area that the cell degraded. In this condition, few GFP-expressing control cells exhibited gelatin degradation (Fig. 1, *A* and *B*). In contrast, overexpression of TRE17 wildtype (WT) dramatically increased the number of cells degrading gelatin, which can be defined by extinction of gelatin fluorescence, whereas TRE17 carrying the C541S mutation that lacks the DUB activity (TRE17/USP-) failed to promote gelatin degradation (Fig. 1, *A* and *B*). These results suggest that TRE17 promotes the ECM degradation by HT-1080 cells in a DUB activity-dependent manner. To examine the specificity of this function of TRE17, we overexpressed another member of the USP family, USP32. *TRE17* gene is derived from the segmental duplication of *USP32* and *TBC1D3*, followed by their fusion (33). Thus, the DUB domain of USP32 is 98% identical to that of TRE17, while USP32 has a distinct N-terminal domain. Even though possessing almost identical DUB domains, only TRE17, but not USP32, could promote the gelatin degradation (Fig. 1, *A* and *B*). This result suggests that the ability to enhance the matrix degradative capacity of HT-1080 cells is specific to TRE17 and not the consequence of a broad effect of DUB overexpression.

We next examined the involvement of endogenous TRE17 in the invasion of HT-1080 cells by knocking down TRE17. The knockdown efficiencies of TRE17 siRNAs were assessed by Western blotting (Fig. S1). The results showed that two independent siRNAs against the *TRE17* gene both suppressed the TRE17 expression at the protein level. Consistent with the results obtained in overexpression experiments, knockdown of endogenously expressed TRE17 in HT-1080 cells suppressed the gelatin degradation: 4 h of incubation allowed the gelatin degradation in control cells, whereas TRE17-knockdown cells exhibited little degradation of gelatin (Fig. 1, *C* and *D*). Similar results were obtained with the other siRNA against TRE17 (Figs. S1 and S2). We also assessed the invasive activity of HT-1080 cells by using Transwell chambers layered with Matrigel, which is composed of the typical matrix that invading cells encounter *in vivo*. The number of cells invaded Matrigel and moved to the bottom side of the chamber was significantly reduced to about 57% of that of control cells by knockdown of TRE17 (Fig. 1, *E* and *F*). Taken together, these results demonstrate that TRE17 enhances the invasive ability of HT-1080 sarcoma cells in a DUB activity–dependent manner.

### TRE17 increases cell surface expression of CD147 and production of MMPs

**Figure 2:**
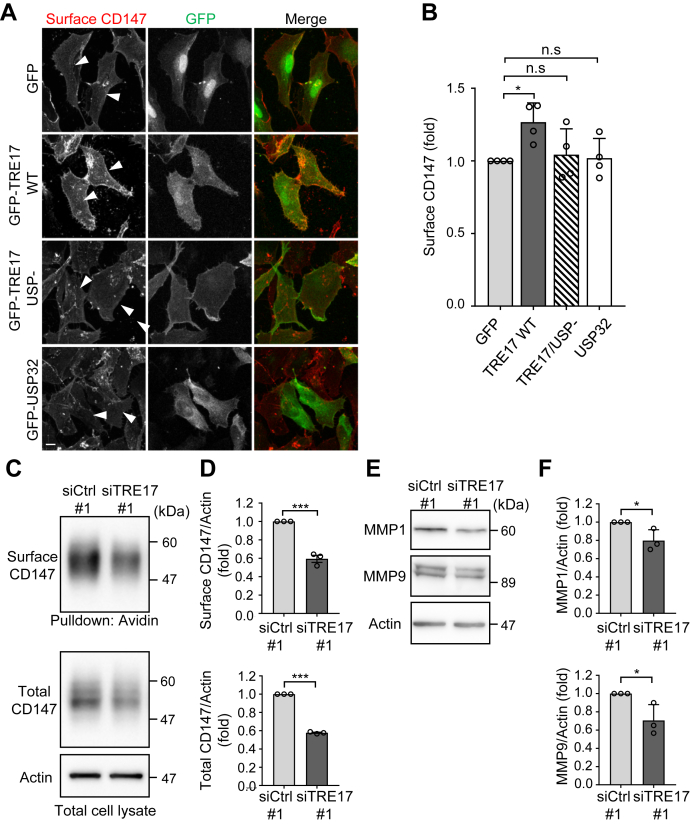
**TRE17 increases cell surface expression of CD147 and production of MMPs.***A*, HT-1080 cells transfected with GFP, GFP-TRE17 WT, GFP-TRE17/USP-, or GFP-USP32 were immunostained with the anti-CD147 antibody without cell permeabilization. *Arrowheads* indicate cells expressing GFP-tagged proteins. Scale bar, 10 μm. *B*, the fluorescent intensities of surface CD147 in *A* were quantified and plotted as the fold change relative to control cells. *C*, cells were transfected with siCtrl #1 or siTRE17 #1, and their surface proteins were isolated by biotin-labeling and Neutravidin-pull-down. Surface proteins and total cell lysates were immunoblotted with the anti-CD147 and anti-actin antibodies. *D*, band intensities of *C* were quantified. The values for CD147 were normalized with those of actin blotting and presented as the fold change compared to the control. *E*, expression levels of MMP1 and MMP9 in cells transfected with siCtrl #1 or siTRE17 #1 were assessed by Western blotting. *F*, band intensities of *E* were quantified as in *D*. All the quantification data are mean ± SD from at least three independent experiments. ∗*p* < 0.05, ∗∗∗*p* < 0.001; n.s., not significant, one-way ANOVA with post hoc Dunnett’s multiple comparison test for *B* and Student’s *t* test for *D* and *F*. MMP, matrix metalloproteinase.

We previously demonstrated that TRE17 promotes the recycling of CIE cargo proteins through deubiquitylating them (4). This finding raises the possibility that TRE17 promotes cell invasion through regulating the recycling of an unknown CIE cargo protein(s), which activates the invasion signaling pathway. Several lines of evidence led us to speculate that CD147 is one of the potential candidate cargoes; first, elevated expression of CD147 as well as TRE17 increases production of MMPs, and CD147 plays a central role in tumor cell invasion and metastasis (15, 22, 23); second, the endocytic recycling determines the cell surface level of CD147, which in turn enhances malignant phenotypes of cancers (27, 28, 29); third, CD147 enters cells *via* CIE and recycled through tubular recycling endosomes specified for CIE cargoes (30, 31). To test this hypothesis, we measured the cell surface level of CD147 by immunofluorescence staining using a monoclonal antibody directed to the extracellular portion of CD147 without cell permeabilization. In TRE17-overexpressing cells, the surface CD147 level was 1.25-times higher than that of control cells (Fig. 2, *A* and *B*). In contrast, overexpression of TRE17/USP- or USP32 did not affect the surface expression of CD147, indicating that TRE17 increases cell surface CD147 depending on its DUB activity. Consistent with this finding, the biochemical analysis, in which cell surface proteins were labeled with biotin, revealed that the knockdown of TRE17 decreases cell surface expression of CD147 (Figs. 2, *C* and *D* and S3, *A* and *B*). Of note, the knockdown of TRE17 also decreased the total CD147 level in this assay. The decrease of both the surface and the total CD147 is likely due to rerouting of the CD147 trafficking from the recycling to the lysosomal degradation pathway, caused by the depletion of TRE17 (described below).

We then analyzed the production of MMPs in this cell line. Treatment of cells with 20 μM AC-73, a specific inhibitor for CD147 (34), decreased the amount of MMP1 and MMP9 (Fig. S3, *C* and *D*), confirming that the CD147-MMPs signaling is conserved in HT-1080 cells as in the majority of cancer cells. In TRE17-knockdown cells, coupled with the reduction of CD147 (Fig. 2, *C* and *D*), the expression levels of MMP1 and MMP9 were 0.8-fold and 0.7-fold lower than those of control cells, respectively (Figs. 2, *E* and *F* and S3, *E* and *F*). Correlated with the decrease of MMPs, the zymography analysis revealed that the MMP9-dependent gelatinolytic activity of HT-1080 cells, which was verified by immunodepletion of MMP9 from the cell lysate (Fig. S4*A*), was significantly suppressed by knockdown of TRE17 (Fig. S4, *B* and *C*). The gelatinolytic activities normalized with the amount of MMP9 protein did not exhibit significant difference between siCtrl and siTRE17 cells (Fig. S4, *B* and *D*), indicating that the reduced invasiveness of TRE17-knockdown cells mainly due to the decreased expression of MMPs but not to the change in enzymatic activities of MMPs. Hence, it appears that the increase of cell surface CD147 mediated by TRE17 enhances the signaling pathway, which induces the production of MMP1 and MMP9, resulting in increased invasiveness of HT-1080 sarcoma cells.

**Figure 3:**
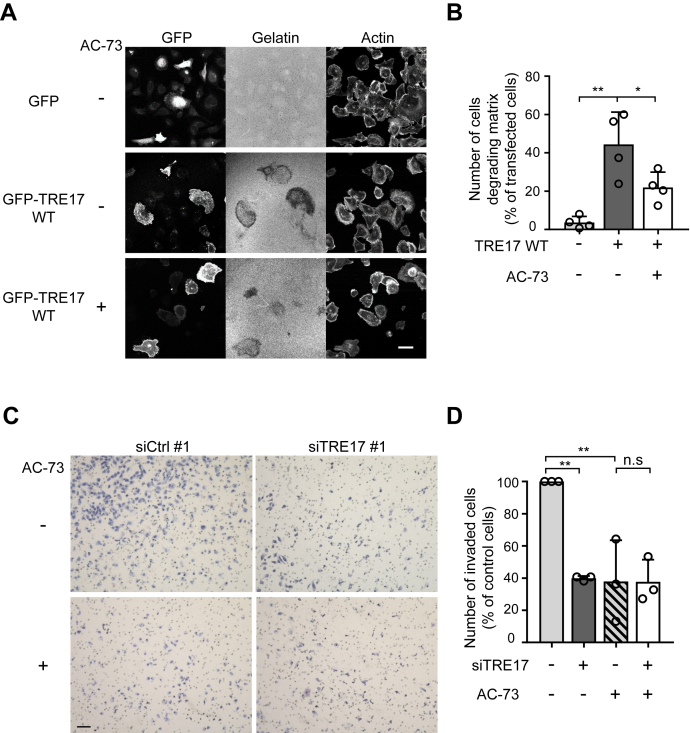
**TRE17 promotes cell invasion through CD147.***A* and *B*, HT-1080 cells transfected with GFP or GFP-TRE17 WT were pretreated with or without 20 μM AC-73 for 6 h and then seeded onto TRITC-gelatin-coated coverslips in the presence or absence of AC-73. Gelatin degradation was analyzed and quantified as in Figure 1, *A* and *B*. Scale bar, 10 μm. *C*, HT-1080 cells transfected with siCtrl #1 or siTRE17 #1 were subjected to the cell invasion assay in the presence or absence of 10 μM AC-73 using transwell chambers as in Figure 1*E*. Scale bar, 100 μm. *D*, the invaded cells were counted in randomly selected ten fields from *C* and plotted as the percentage of control cells (siCtrl without AC-73). The graphs shown are mean ± SD from at least three independent experiments. ∗*p* < 0.05, ∗∗*p* < 0.005, n.s., not significant, one-way ANOVA with post hoc Tukey’s multiple comparison test.

To examine whether CD147 is responsible for the TRE17-dependent promotion of cell invasion, we assessed the invasiveness of HT-1080 cells under the combination of TRE17 overexpression and pharmacological inhibition of CD147. A marked increase of the matrix degradative activity of the cells caused by TRE17 overexpression was attenuated by the pharmacological inhibition of CD147 (Fig. 3, *A* and *B*), indicating that TRE17 depends on CD147 to promote matrix degradation.

We also tested involvement of CD147 in TRE17-regulated invasion by a Matrigel invasion assay. As observed in Figure 1, *E* and *F*, knockdown of TRE17 reduced the number of invaded cells in the absence of the CD147 inhibitor (Fig. 3, *C* and *D*). Similarly, pharmacological inhibition of CD147 suppressed the cell invasiveness to a comparable level as that of TRE17-knockdown cells without AC-73 treatment. However, knockdown of TRE17 did not further suppress the cell invasion under the presence of the CD147 inhibitor, suggesting that TRE17 promotes cell invasion through CD147. Taken together, these results strongly support the hypothesis that TRE17 promotes tumor cell invasion by increasing the CIE cargo CD147, which induces the production of MMPs.

### TRE17 suppresses targeting of CD147 to lysosomes in a DUB activity–dependent manner

**Figure 4:**
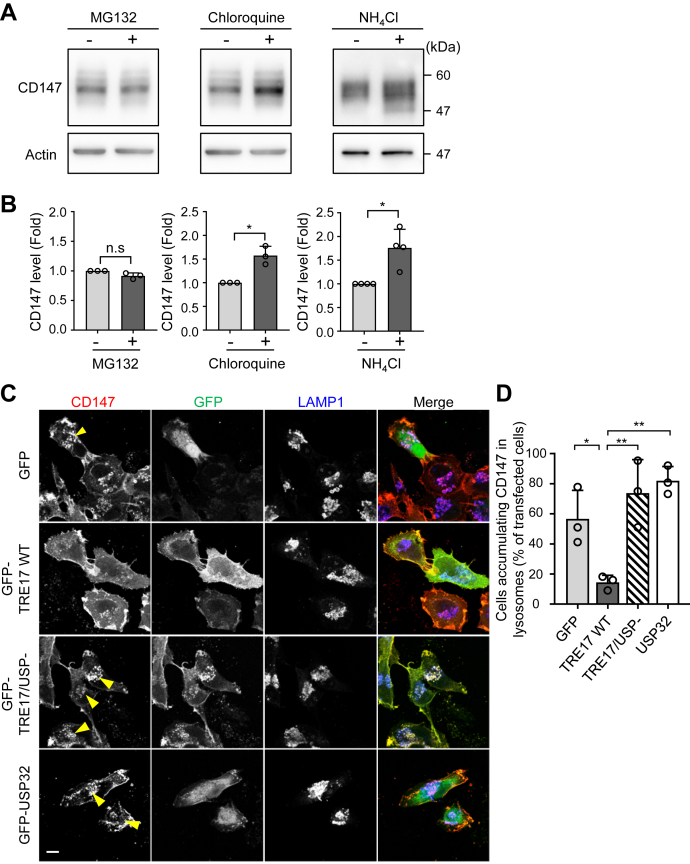
**TRE17 prevents CD147 from trafficking to lysosomes for degradation.***A*, HT-1080 cells were treated with or without 10 μM MG132, 25 μM chloroquine, or 25 mM NH_4_Cl, and cell lysates were subjected to Western blotting using indicated antibodies. *B*, band intensities in *A* were quantified and plotted as the fold change compared to the control cells. *C*, HT-1080 cells were transfected with GFP, GFP-TRE17 WT, GFP-TRE17/USP-, or GFP-USP32. After 24 h, anti-CD147 antibody was added to cells and allowed to bind to CD147 and be internalized for 1 h. Cells were washed and transferred to the medium containing 25 mM NH_4_Cl and cultured for 16 h. Cells were also co-stained with anti-Lamp1 antibody. *Arrowheads* indicate transfected cells accumulating CD147 at lysosome compartments. Scale bar, 10 μm. *D*, transfected cells in *C* were randomly selected (50–60 cells), and the number of cells showing accumulation of CD147 at lysosomes was counted and plotted as the percentage of transfected cells. All the graphs shown are mean ± SD from at least three independent experiments. ∗*p* < 0.05, ∗∗*p* < 0.005; n.s., not significant, one-way ANOVA with post hoc Tukey’s multiple comparison test.

The surface CD147 level is determined by the balance between intracellular transport *via* the ER–Golgi biosynthetic pathway and the rate of protein turnover (2, 35). In general, membrane proteins and cytosolic proteins are tagged with a ubiquitin chain, which leads to degradation by lysosomes and the proteasome, respectively. Since TRE17 requires its DUB activity to enhance cell invasiveness and to increase cell surface CD147 (Figs. 1 and 2), we explored the degradation pathway of CD147. Treatment with the lysosome inhibitors chloroquine and NH_4_Cl, but not the proteasome inhibitor MG132, significantly increased the expression level of CD147 (Fig. 4, *A* and *B*), suggesting that the turnover of CD147 is predominantly controlled by the lysosomal degradation and this pathway contributes to the regulation of the CD147 abundance.

We next examined whether TRE17 affects intracellular trafficking of CD147 to lysosomes. To chase CD147 intracellular trafficking, cells were incubated with anti-CD147 antibody for 1 h, and the fate of antibody-bound CD147 was analyzed. To visualize cargo delivery to lysosomes, cells were treated with NH_4_Cl to neutralize the lysosomal degradation, resulting in the accumulation of lysosome-targeted proteins. After 16 h of incubation, most of the control cells (nearly 60%) exhibited a clear accumulation of CD147, which was co-stained with the late endosome/lysosome marker Lamp1 (Fig. 4, *C* and *D*). Meanwhile, in cells overexpressing TRE17 WT, the accumulation of CD147 at the lysosomal compartment was significantly suppressed (less than 15% of TRE17-expressing cells exhibited CD147 accumulation), and CD147 remained mostly at the plasma membrane and the cytoplasm. This inhibitory effect was not observed in cells overexpressing TRE17/USP- or USP32 (Fig. 4, *C* and *D*). These results indicate that TRE17 prevents trafficking of CD147 to lysosomes in a DUB activity-dependent manner.

**Figure 5:**
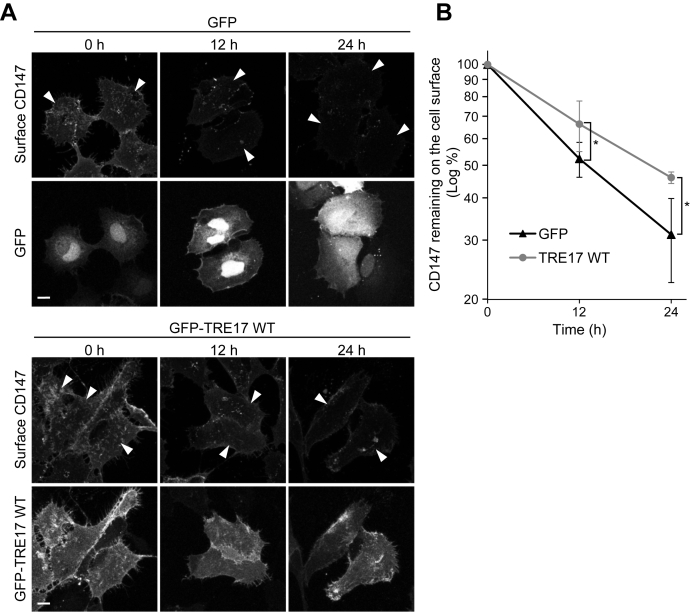
**TRE17 stabilizes surface CD147.***A*, HT-1080 cells transfected with GFP or GFP-TRE17 WT were incubated with anti-CD147 antibody at 4 °C for 1 h to label cell surface CD147. Cells were then incubated at 37 °C for 0, 12, and 24 h. Antibody-bound CD147 proteins remained at the cell surface were detected by the secondary antibody. *Arrowheads* indicate cells transfected with GFP-tagged proteins. Scale bar, 10 μm. *B*, fluorescence intensities of surface CD147 in *A* were measured and plotted as the percentage of the 0 h time point. Shown are mean ± SD from four independent experiments. ∗*p* < 0.05, Student’s *t* test.

Suppression of CD147 transport to lysosomes indicates rerouting of CD147 to the recycling pathway, which eventually results in the prolonged half-life of CD147 on the cell surface. To test this hypothesis, the pulse-chase analysis of surface CD147 was performed. The cell surface CD147 proteins were pulse-labeled by incubating cells with anti-CD147 antibody at 4 °C for 1 h, followed by further incubation for 12 and 24 h at 37 °C. During this incubation, the labeled CD147 proteins undergo several rounds of recycling before being targeted to lysosomal degradation. After incubation, the antibody-labeled CD147 proteins remaining at the cell surface were detected by indirect immunofluorescence staining without cell permeabilization. In this assay, the level of surface CD147 was gradually reduced over time, reflecting the turnover of the pulse-labeled CD147 proteins on the cell surface (Fig. 5). In TRE17 WT-overexpressing cells, the decline of surface CD147 was slower than that in control cells, supporting the hypothesis that TRE17 prolongs the half-life of cell surface CD147. Staining of the total pulse-labeled CD147 proteins by permeabilizing the cells also confirmed that degradation of CD147 is delayed in cells overexpressing TRE17 (Fig. S5). These results suggest that TRE17 prolongs the half-life of cell surface CD147 by altering the sorting events from lysosomal degradation to the recycling pathway.

### CD147 is deubiquitylated by TRE17

**Figure 6:**
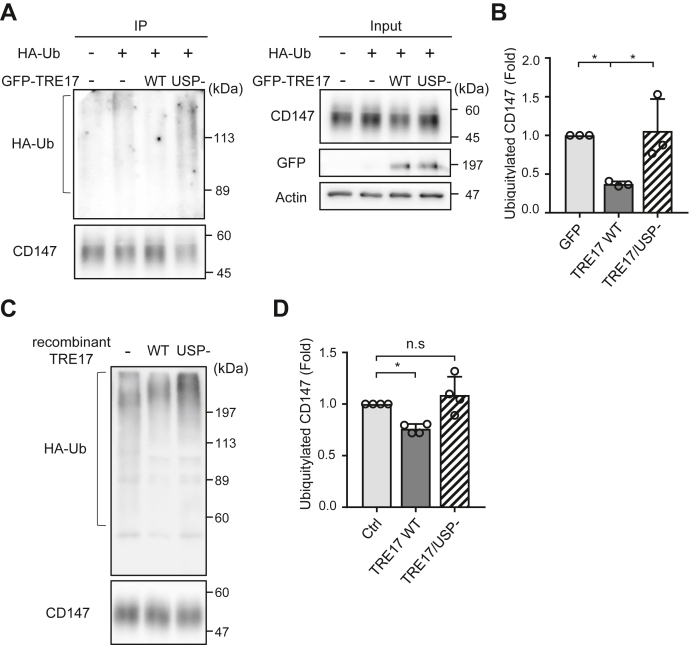
**TRE17 promotes deubiquitylation of CD147.***A*, HT-1080 cells were transfected with GFP, GFP-TRE17 WT, or GFP-TRE17/USP- together with or without HA-Ubiquitin as indicated. After 24 h post transfection, endogenous CD147 was immunoprecipitated with anti-CD147 antibody from denatured cell lysates. Cell lysates and precipitates were separated by SDS-PAGE and immunoblotted with the antibodies as indicated. *B*, the intensities of ubiquitylated CD147 that are indicated by the bracket in the anti-HA blot in *A* were measured and normalized to the respective precipitated CD147 level. The values are plotted as the fold change from the cells transfected with GFP and HA-Ubiquitin. *C*, HEK293T cells were transfected with HA-Ubiquitin. After 24 h post transfection, cells were treated with 20 μM of PR619 for 2 h and endogenous CD147 was immunoprecipitated with anti-CD147 antibody. The precipitate was divided into aliquots and incubated with recombinant TRE17 proteins as indicated for 2 h. Ubiquitin remaining on CD147 was detected by anti-HA immunoblotting. *D*, ubiquitylation of CD147 was quantified as in *B*. The values are plotted as the fold change from the control. Shown are mean ± SD from at least three independent experiments. ∗*p* < 0.05, one-way ANOVA with post hoc Tukey’s multiple comparison test.

Because the DUB activity of TRE17 is required for altering the itinerary of CD147 trafficking (Fig. 4, *C* and *D*) and promoting cell invasion (Fig. 1, *A* and *B*), these functions of TRE17 are likely to be mediated by deubiquitylation of CD147. We therefore tested the ubiquitylation level of CD147. CD147 was immunopurified with anti-CD147 antibody from the cell lysates obtained from cells expressing HA-ubiquitin and either GFP-TRE17 WT or USP-. To ensure that HA-ubiquitin signals were derived from CD147 and not copurified proteins, the cell lysates were denatured by SDS and boiling before the pull-down. In control cells expressing GFP, smeared bands of HA-ubiquitin, which represents ubiquitylated CD147, were observed (Fig. 6*A*). These smeared bands were remarkably decreased in cells overexpressing TRE17 WT, whereas TRE17/USP- did not affect ubiquitylation of CD147. The levels of ubiquitylation were quantified by normalizing the intensities of HA-ubiquitin to the amount of immunopurified CD147. TRE17 WT-overexpressing cells exhibited approximately 50% reduction in the ubiquitylated CD147 level compared to that of control cells (Fig. 6*B*). In contrast, cells expressing TRE17/USP- showed a comparable level of ubiquitylated CD147 as the control cells (Fig. 6*B*).

To test whether TRE17 is capable of directly deubiquitylating CD147 *in vitro*, we prepared the N-terminal-deleted recombinant TRE17 protein (amino acids 526–1406) from *Escherichia coli* (*E. coli*) as previously described in three independent studies (16, 17, 18), in which the authors performed *in vitro* deubiquitylation assays with N-terminal-deleted TRE17. Ubiquitylated CD147 was immunopurified from HEK-293T cells expressing HA-Ub using anti-CD147 antibody and incubated with the recombinant TRE17 proteins. The TRE17 WT protein efficiently deubiquitylated CD147 and the ubiquitin level of CD147 exhibited approximately 25% reduction compared to the control, whereas incubation with the TRE17/USP- protein did not change the ubiquitin level of CD147 (Fig. 6, *C* and *D*). These results demonstrate that TRE17 promotes deubiquitylation of CD147 through its DUB activity.

## Discussion

**Figure 7:**
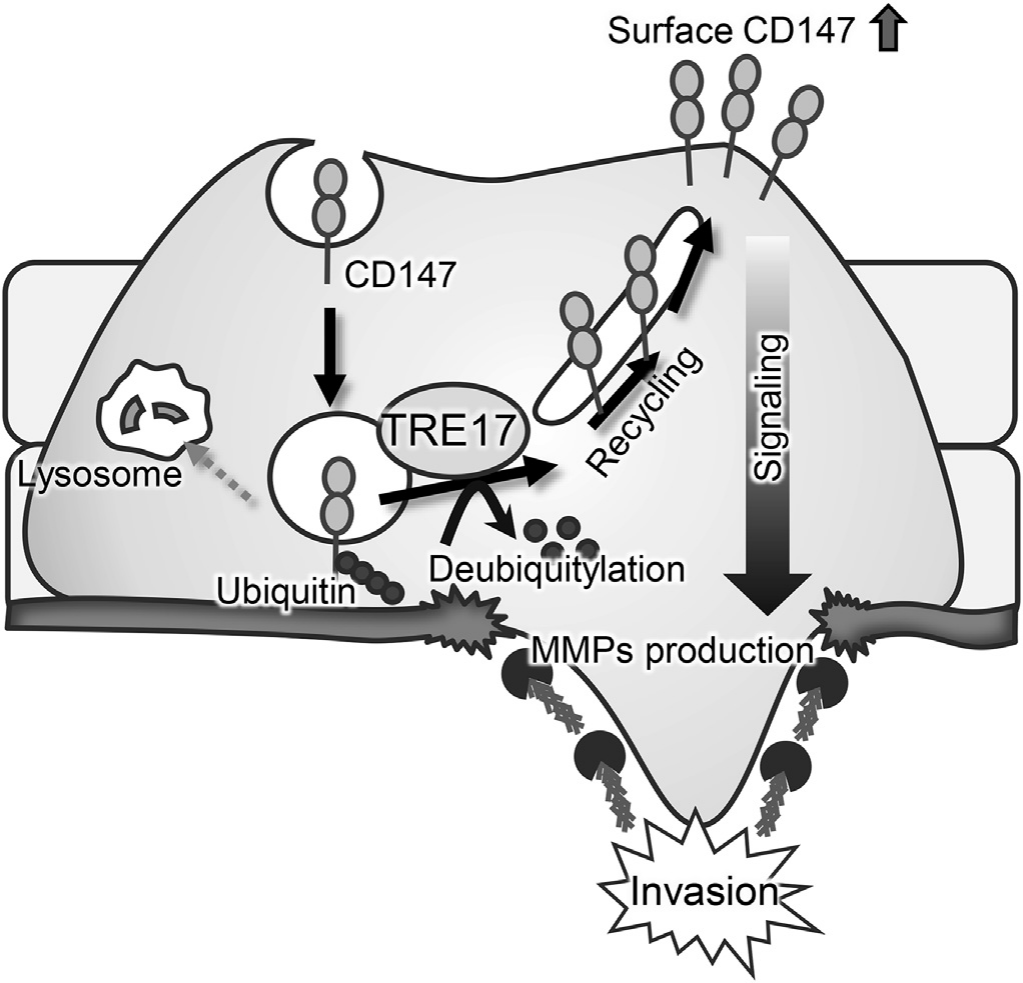
**A model for TRE17-mediated promotion of tumor cell invasion.** Increased expression of TRE17 promotes deubiquitylation of CD147, which prevents CD147 from trafficking to lysosomes, resulting in increase of cell surface CD147. Increase of surface CD147 in turn enhances the signal to induce MMP production, leading to promotion of cell invasion. MMP, matrix metalloproteinase.

The present study identifies CD147 as a target CIE cargo of TRE17, which is responsible for TRE17-mediated promotion of cell invasion. We show in the sarcoma-derived cell line, HT-1080 cells, that TRE17 prevents CD147 from targeting to lysosomal degradation by deubiquitylating it, resulting in the increase of cell surface CD147. This increase in turn facilitates MMP synthesis, thereby promoting cell invasion. Our findings propose a model for the pathogenesis of neoplastic diseases driven by elevated expression of TRE17 (Fig. 7).

It has been well characterized that high expression of CD147 at the cell surface is strongly associated with tumor progression in many types of cancer. In addition to its biosynthesis, the protein stability and intracellular trafficking determine the surface level of CD147. Several types of posttranslational modification, including glycosylation and ubiquitylation, are known to contribute to these processes. As with other immunoglobulin superfamily members, CD147 is highly glycosylated, and the *N*-glycosylation of CD147 is required for its maturation and localization to the plasma membrane (35). Recent studies have identified several ubiquitin E3 ligases targeting CD147 and demonstrated that ubiquitylation mediated by these ligases is associated with the surface level and stability of CD147, which contribute to tumor cell invasion and malignancies (36, 37). On the other hand, the DUB that targets CD147 has not been identified to date. Our present study identifies the first DUB regulating the trafficking and stability of CD147. It appears that reversible ubiquitylation of CD147 mediated by a ubiquitin ligase and TRE17 plays a pivotal role in the control of CD147 abundance and tumor cell invasion.

CIE cargo proteins undergo two different trafficking routes to their final destinations. Conventional CIE cargo proteins (such as MHCI, CD55, CD59, Glut1, and Tac) travel along the typical CIE route, which is characterized by localization of the cargo to Rab5- and EEA1-positive endosomes (38). From there the cargo is either targeted to lysosomal degradation or recycled back to the plasma membrane. Another group of CIE cargo proteins (CD44, CD98, and CD147) is predominantly recycled back to the plasma membrane in a process dependent on Rab22A, leading to the prolonged surface half-life of these cargo proteins (30). Eyster *et al.* (3) have shown that MARCH E3 ubiquitin ligases target most of these CIE cargoes from both groups except for the GPI-anchored proteins CD55 and CD59, which lack the cytoplasmic domain. However, CD147 was not targeted by MARCH family members, even though CD147 possesses the cytoplasmic domain with several potential lysine residues for ubiquitylation. Nevertheless, the results obtained in the current study suggest that CD147 undergoes basal ubiquitylation in HT-1080 cells, and overexpression of TRE17 deubiquitylates it to alter the itinerary of CD147 (Figs. 4 and 6). These findings raise the question of which ubiquitin ligase(s) is the counterpart of TRE17 to target CD147 to lysosomes. Given that there are more than ten members of the MARCH family, other MARCH members that have not been tested in the previous study could be the candidates. Another potential candidate is the F-Box protein FBXO22. The recent study has shown that FBXO22 ubiquitylates CD147 and promotes its degradation, although whether this ubiquitylation targets CD147 to lysosomes remains elusive (36). This question has to be answered in future studies, and it is of interest to analyze the balance of these ubiquitin ligases and TRE17 in neoplasms associated with dysregulation of CD147.

Besides its key role in protein degradation, ubiquitylation also regulates endocytosis, localization, and activity of the substrate proteins. This is also the case for CD147, and it has been reported that the ubiquitin ligase TRAF6 ubiquitylates CD147 to facilitate its recruitment to the plasma membrane without affecting its stability, leading to the promotion of melanoma invasion and metastasis (37). There are several potential lysine residues in the cytoplasmic domain of CD147 that could be ubiquitylated (37). Selective ubiquitylation of these lysine residues and/or combination of them might diversify the regulation of CD147. It is also known that different types of ubiquitin linkages have distinct functions. For instance, Lys48-linked chains are the typical tag for proteasomal degradation; Lys63-linked chains regulate intracellular localization and trafficking to lysosomes; Lys29-linked chains also target the substrate for lysosomal degradation (39, 40). CD147 may undergo several types of ubiquitin linkages, conferring the complexity of ubiquitylation-mediated regulation. Furthermore, the ubiquitylation-deubiquitylation cycle is spatiotemporally regulated in the cell, and thus when and where CD147 is ubiquitylated might determine the fate of ubiquitylated CD147. These diverse functions of ubiquitylation might enable fine-tuning of the stability, localization, and activity of CD147.

TRE17 was originally identified from Ewing sarcoma as an oncogene (5), and an increasing amount of evidence supports the pro-oncogenic function of TRE17. High expression of TRE17 is often observed in benign but locally aggressive mesenchymal neoplasms, including ABC and NF, and associated with tumorigenesis of these types of cells (8, 11, 15, 19). Moreover, several studies demonstrated that TRE17 is also associated with various malignant cancers, such as breast cancer and colorectal cancer, and promotes their invasion and metastasis (5, 12, 13, 14). Several signaling pathways contributing to the oncogenesis of TRE17 have also been identified: TRE17 increases the production of MMPs and promotes tumorigenesis of osteosarcoma cells through the activation of NF-κB and Jack1-STAT3 pathways, respectively (15, 16, 17, 18). To our surprise, however, the recent studies from Chou *et al.* suggested the tumor-suppressive function of TRE17 in Ewing sarcoma. They showed that TRE17 sensitizes Ewing sarcoma cells to interferon (IFN) stimulation by stabilizing the Jak1 kinase, which induces apoptosis of cancer cells (41). Furthermore, the increase in IFN responses by TRE17 suppresses the tumorigenesis of Ewing sarcoma by increasing the secretion of cytokines from tumor cells, which promotes the infiltration of immune cells (42). It is interesting to note that the enhancement of IFN responses is likely due to the increase of surface IFN receptors. Although the intracellular trafficking mechanism of IFN receptors is not yet clarified, it is of interest to investigate the role of TRE17 in this process. Collectively, it is suggested that TRE17 is a multifunctional DUB with both pro- and anti-oncogenic potentials. It is speculated that TRE17’s pro- or anti-oncogenic function may depend on whether TRE17 acts as a driver gene or is involved in the later processes. The combination of mutations in other genes and/or differences in the microenvironments may also influence the dominant effect of TRE17 on oncogenesis. Nonetheless, our present study clearly demonstrates the involvement of the TRE17-CD147 axis in sarcoma cell invasion, one of the main features of TRE17-driven neoplasms. The finding of this novel signaling pathway will provide insights into the complex mechanisms of TRE17-mediated oncogenesis.

## Experimental procedures

### Plasmids and siRNAs

Plasmids for expression of GFP-TRE17, GFP-TRE17/USP-, and GFP-USP32 were prepared as described previously (4). To construct a plasmid for expression of the GST-tagged recombinant N-terminal-deleted TRE17, a DNA fragment encoding 529 to 1406 amino acids of TRE17 WT or USP- was amplified with PCR and subcloned into the pGEX-6P-1 vector (Cytiva). For knockdown experiments, two independent sets of siRNAs, Stealth RNAi siRNAs (siControl #1, 12935300; siTRE17 #1, 10620318) and On-TARGET plus siRNAs (siControl #2, D-001810-01; siTRE17 #2, J-006096-13) were purchased from Thermo Fisher Scientific and Horizon Discovery, respectively.

### Antibodies

Mouse monoclonal antibodies used in this study were purchased from the following companies: anti-CD147 (306202) from BioLegend, anti-MMP1 (sc-58377) from Santa Cruz, anti-GFP (04363-24) from Nacalai tesque, anti-HA (MMS-101P) from Covance Research Products, and anti-Actin (A5441) from Sigma-Aldrich. Rabbit polyclonal anti-TRE17/USP6 (ab179751) and anti-LAMP1 (ab24170) antibodies were purchased from Abcam, and goat polyclonal anti-MMP9 (AF911) antibody was purchased from R&D Systems. All Alexa-Fluor-conjugated secondary antibodies were purchased from Invitrogen Molecular Probes.

### Cell culture and transient transfection of plasmid DNAs and siRNA

HT-1080 cells and HEK293T cells were cultured in Dulbecco’s Modified Eagle’s medium (DMEM) containing 4.5 g/l glucose and 2 mM glutamine (Nacalai tesque) supplemented with 10% fetal bovine serum (FBS; Life Technologies) and 1% Penicillin/Streptomycin (Nacalai tesque), and grown under a 5% CO_2_ atmosphere at 37 °C. Cells were transfected with plasmid DNAs using Lipofectamine 2000 (Life Technologies) or Polyethylenimine Max (COSMO Bio) or with siRNAs using lipofectamine 2000 or Lipofectamine RNAi Max (Life Technologies) according to the manufacturers’ instructions.

### Assessment of knockdown efficiencies of siRNAs

The knockdown efficiencies of TRE17 siRNAs were analyzed by Western blotting for co-transfected GFP-TRE17 and endogenous TRE17. At 24 h post siRNA transfection, cells were transfected with a plasmid encoding GFP-TRE17 and further incubated for 48 to 72 h. The cells were harvested and subjected to Western blotting as described below. For measurement of endogenous TRE17 protein, cells were harvested at 48 h post siRNA transfection and subjected to Western blotting.

### Assay to measure gelatin degradation

Coverslips coated with TRITC-labeled gelatin were prepared as described previously (43). HT-1080 cells transfected with GFP-tagged proteins were plated on the coverslips and incubated for 2 to 5 h to allow them to degrade fluorescent gelatin. The cells were then fixed with 4% PFA, permeabilized with 0.1% Triton X-100, and incubated with Alexa Fluor 633 Phalloidin (Thermo Fisher Scientific) for 20 min. The number of transfected cells and cells degrading gelatin were counted under the fluorescence microscope KEYENCE BZ-X710. More than 100 cells were counted in each experiment, and the ratio of cells degrading gelatin to transfected cells was calculated. In the experiment, in which TRE17 was knockdowned, cells were stained with 4,6-diamidino-2-phenylindole (Thermo Fisher Scientific) for 20 min. The gelatin-degraded area was measured with the ImageJ software and normalized with the total cell number.

### *In vitro* cell invasion assay

Cell invasion was assessed using BioCoat Matrigel Invasion Chambers (BD) according to the manufacture’s instructions. Briefly, 2.5 × 10^4^ cells were plated onto the upper chamber of BioCoat Matrigel Invasion Chamber containing a polycarbonate transwell filter coated with Matrigel and incubated at 37 °C for 22 h. Cells that remained on the upper surface of the filter were removed using cotton swabs. Cells that had moved to the lower membrane surface were fixed in 4% paraformaldehyde, stained with hematoxylin, and counted using the ImageJ software (ten random fields per well at 100× magnification).

### Western blotting

The cell lysates or pull-downed proteins were separated by SDS-PAGE and transferred onto a PVDF membrane. The membrane was blocked with 5% nonfat milk for 1 h at RT and incubated with the specified antibodies at 4 °C overnight. Membranes were subsequently incubated with HRP-conjugated secondary antibodies (Cell Signaling Technology) at RT for 1 h. The signals were detected by the luminescent image analyzer LAS-4000 mini (Fujifilm).

### Biotin labeling–based assessment of surface CD147

HT-1080 cells were incubated with EZ-linked NHS-SS-Biotin (0.5 mg/ml in PBS) at 4 °C for 1 h to label the surface proteins. Unbound biotin was removed by washing cells with PBS twice and quenched by washing cells with 50 mM NH_4_Cl/PBS for 10 min. Cells were washed with PBS and solubilized with 500 μl of the lysis buffer [50 mM Tris-HCl, pH 7.5, 150 mM NaCl, 1% Triton X-100, 1 mM EDTA, 0.1% SDS, and protease inhibitor cocktail (Nacalai Tesque)] for 20 min at 4 °C. The cell lysate was centrifuged, and the supernatant was incubated with 20 μl of NeutrAvidin agarose (Thermo Fisher Scientific) for 6 h at 4 °C to precipitate all biotinylated proteins. After washing four times with the lysis buffer, proteins bound to the beads were eluted with the SDS-PAGE sample buffer by boiling, and Western blotting was performed as described above.

### Zymography analysis

HT-1080 cells transfected with siRNAs were solubilized in the zymography lysis buffer [50 mM Tris-HCl, pH 7.5, 150 mM NaCl, 10% glycerol, 1% Triton X-100, and protease inhibitor cocktail (Nacalai Tesque)] and centrifuged. SDS sample buffer without reducing agents was added to the supernatant and incubated at room temperature for 30 min. Samples were run on an 8% acrylamide SDS-PAGE gel containing 1 mg/ml of gelatin, and the gel was incubated with 2.5% Triton-X-100 at room temperature for 20 min twice. The gel was equilibrated in the developing buffer (50 mM Tris-HCl, pH 7.5, 200 mM NaCl, 5 mM CaCl_2_, 0.02% Brij35, and 1 μM ZnCl_2_) for 30 min at room temperature, and the buffer was replaced to a fresh developing buffer. The gel was further incubated at 37 °C for 20 h and then subjected to Coomassie brilliant blue staining.

### Immunodepletion of MMP9 from the cell lysate

HT-1080 cells were solubilized in the zymography lysis buffer as described above and divided into equal amounts, one of which was immediately added with SDS sample buffer without reducing agent. The anti-MMP9 (R&D systems) antibody or control IgG and protein G agarose (Thermo Fisher Scientific) were added to the remaining replicate samples and incubated at 4 °C for 2 h. The samples were centrifuged, and the supernatants were collected and subjected to a zymography analysis as describe above.

### Purification of recombinant TRE17 proteins

For an *in vitro* deubiquitylation assay, we prepared the recombinant N-terminal-deleted TRE17 (amino acids 529–1406) protein as previously reported (16, 17, 18), because of the low protein yield and the low DUB activity of the full-length TRE17 protein. *E. coli* BL21(DE3) transformed with pGEX-6P-1/TRE17 (529–1406) WT or USP- was cultured in the LB medium and treated with 0.2 mM of isopropyl β-D-thiogalactopyranoside for 20 h at 18 °C. Cells were harvested and resuspended in the GST-lysis buffer (50 mM Tris-HCl, pH 7.5, 150 mM NaCl, 1% Triton X-100, 1 mM EDTA, 1 mM dithiothreitol, 1 mM phenylmethylsulfonyl fluoride, 1 μg/ml Pepstatin A, and 2 μg/ml Aprotinin) and treated with 0.5 mg/ml lysozyme for 30 min on ice. After sonication, the lysate was rotated at 4 °C for 30 min and centrifuged. COSMOGEL GST-accept (Nacalai Tesque) was added to the supernatant and incubated at 4 °C for 2 h. The beads were washed three times with the GST-lysis buffer, followed by washing with the DUB assay buffer (20 mM Tris-HCl pH 7.5, 100 mM NaCl, 0.05% Tween-20, and 5 mM β-mercaptoethanol) three times. Recombinant proteins were recovered from the beads by digesting the GST-tag by Turbo 3C protease (Accelagen).

### Analysis of ubiquitylated CD147

For the detection of ubiquitylated CD147, HT-1080 cells plated on a 6 cm dish were transfected with plasmid DNAs and incubated for 24 h. Cells were rinsed three times with PBS, harvested, and pelleted at 300 *g*. Cell pellets were solubilized in 50 μl of the lysis buffer [50 mM Tris-HCl, pH 7.4, 0.25 M NaCl, 0.1% Triton X-100, 1 mM EDTA, 0.2 mM dithiothreitol, 0.8 mM N-ethylmaleimide, 0.2 mM iodoacetamide, and protease inhibitor cocktail (Nacalai Tesque)] and incubated on ice for 5 min. The cell extract was denatured by adding SDS to the final concentration of 1% and boiled for 15 min. The SDS was quenched by adding 50 μl of 10% Triton X-100 and 450 μl of lysis buffer without protease inhibitors and placed on ice for 30 min. The lysate was centrifuged at 13,000*g* for 5 min, and 15 μl of Protein G Agarose (Thermo Fisher Scientific) and 1 μl of the anti-CD147 antibody solution (Biolegend) were added to the supernatant. The lysate was rocked for 2 h at 4 °C, and the beads were washed four times with the lysis buffer without protease inhibitors. The beads were boiled for 10 min with the SDS-PAGE sample buffer to elute the bound proteins. The eluate was separated by SDS-PAGE and immunoblotted as described above.

For *in vitro* DUB assays, HEK293T cells plated on a 6 cm dish were transfected with HA-Ub for 24 h and treated with 20 μM of PR619, a general inhibitor of DUBs, for 2 h. Cells were harvested and solubilized in 600 μl of the extraction buffer [50 mM Tris-HCl, pH 7.5, 0.15 M NaCl, 1% Triton X-100, 10% glycerol, 0.8 mM N-ethylmaleimide, 0.2 mM iodoacetamide, and protease inhibitor cocktail (Nacalai Tesque)] and centrifuged. The supernatant was then subjected to immunoprecipitation as described above. The beads were washed twice with the extraction buffer containing 0.5 M NaCl without protease inhibitors and three times with the DUB assay buffer. Then, the beads bound with ubiquitylated CD147 were divided into equal portions and incubated with the DUB assay buffer with or without TRE17 recombinant proteins for 2 h at 37 °C. The beads were washed with the DUB assay buffer three times and boiled with the SDS-PAGE sample buffer. The eluate was separated by SDS-PAGE and then immunoblotted with anti-HA antibody.

### Immunofluorescence staining for CD147

HT-1080 cells were seeded on coverslips and transfected with plasmid DNAs, which express GFP-tagged proteins. After 24 h of incubation, cells were fixed for 10 min in 2% formaldehyde in PBS and rinsed with PBS. Cells were then blocked with 10% FBS in PBS and incubated with primary antibodies in PBS containing 10% FBS without cell permeabilization. Alexa-Fluor–conjugated secondary antibodies were used to detect the primary antibodies. To quantify the surface level of CD147, 15 to 25 transfected cells were selected, and their z-stack confocal images were obtained using TCS-SP8 (Leica Microsystems) or LSM700 (Carl Zeiss) confocal microscopes. The CD147 signal intensities were measured by the Fiji software (NIH) and normalized with the cell areas. For pulse-labeling and chase of the surface CD147, HT-1080 cells seeded on coverslips were incubated with DMEM containing 10% FBS and anti-CD147 antibody at 4 °C for 1 h. After washing cells with ice-cold DMEM without FBS to remove unbound antibodies, a set of cells were subjected to fixing and staining with Alexa Fluor-conjugated secondary antibodies with or without cell permeabilization using 0.2% saponin to detect total or surface pulse-labeled CD147, respectively. The rest of the cells were incubated with DMEM containing 10% FBS at 37 °C for the indicated time, fixed, and then CD147-antibody complexes remained were stained with Alexa Fluor-conjugated secondary antibodies with or without cell permeabilization. The surface or total CD147 levels were quantified as described above.

### Assay to analyze intracellular trafficking of CD147

HT-1080 cells were transfected with plasmid DNAs, which express GFP-tagged proteins, and cultured for 20 to 24 h. Cells were preincubated with the medium containing anti-CD147 antibody (2.5 μg/ml) for 1 h at 37 °C. After washing with the medium, cells were transferred to the fresh medium containing 25 mM NH_4_Cl and cultured for 16 h. Cells were subjected to fixing and blocking and then incubated with anti-GFP and anti-Lamp1 antibodies in PBS containing 10% FBS with 0.2% saponin. Alexa-Fluor-conjugated anti-mouse IgG antibody was used to visualize anti-CD147 antibody. Images were obtained by a confocal microscope as described above. To quantify the transport of CD147 to lysosomes, 30 to 60 transfected cells were selected, and the number of cells accumulating CD147 at lysosomes was counted.

### Statistical analyses

All quantified data were expressed as means ± SD and analyzed by Student’s *t* test or one-way ANOVA with post hoc Tukey’s or Dunnett’s test using the Prism 8 software (GraphPad).

## Data availability

The data that support the findings of this study are available from the corresponding author (Y.F.), upon reasonable request.

## Supporting information

This article contains supporting information.

## Conflict of interest

The authors declare that they have no conflict of interest with the contents of the article.
