# Supplementary material for: Ubiquitin-specific protease TRE17/USP6 promotes tumor cell invasion through the regulation of glycoprotein CD147 intracellular trafficking
Source: J Biol Chem. 2022 Aug 2;298(9):102335. doi: 10.1016/j.jbc.2022.102335 (PMC9440431; doi:10.1016/j.jbc.2022.102335)
Supplement: Supplemental Figures S1–S5 [file mmc1.pdf]

Supporting information

Ubiquitin-specific protease TRE17/USP6 promotes tumor cell invasion through the regulation of glycoprotein CD147 intracellular trafficking

Yukino Ogura, Norihiko Ohbayashi, Yasunori Kanaho,
Atsushi Kawaguchi, Yuji Funakoshi

List of the contents

Figure S1……………………………………Page S-2
Figure S2……………………………………Page S-3
Figure S3……………………………………Page S-4
Figure S4……………………………………Page S-5
Figure S5……………………………………Page S-6

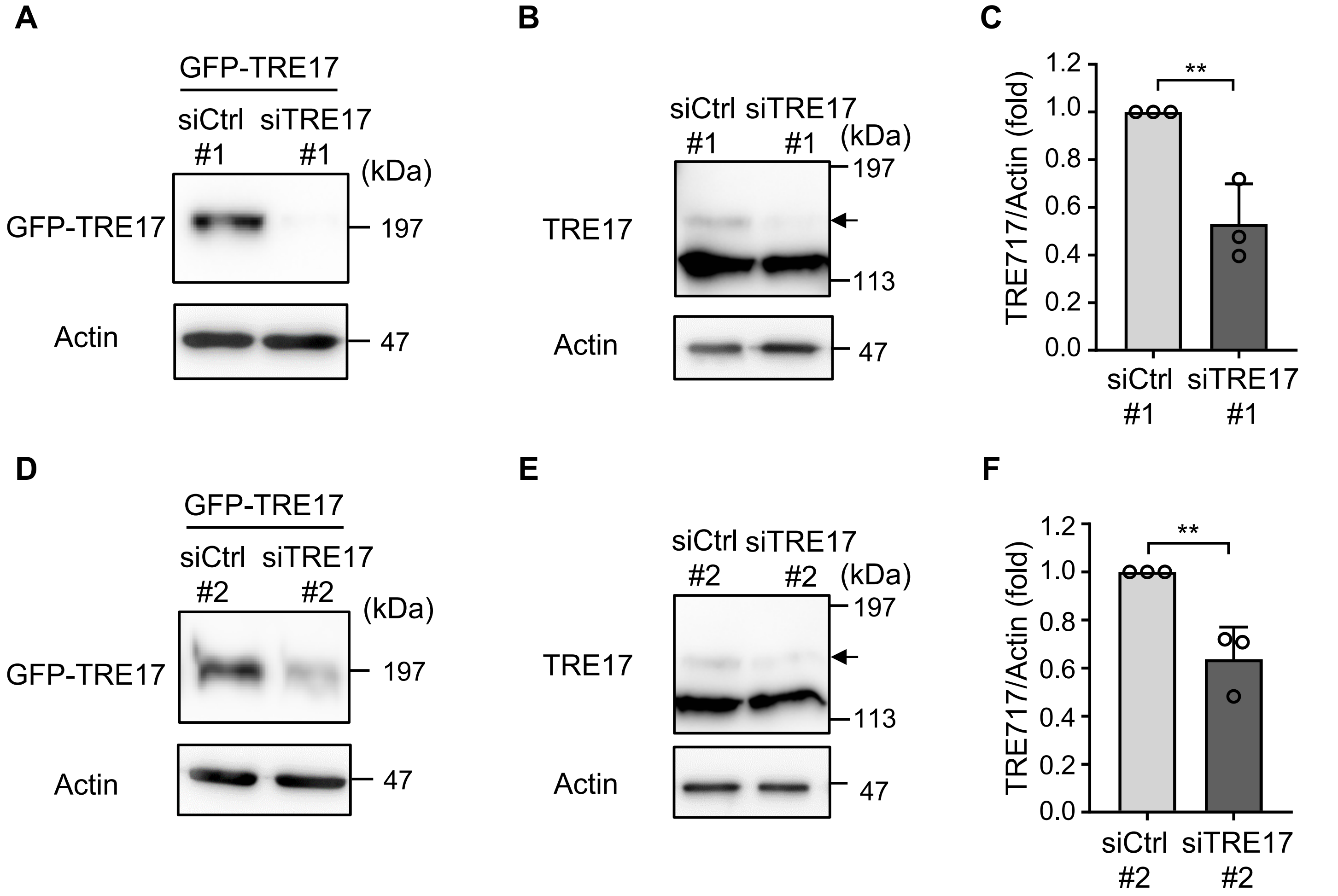

Figure S1. Knockdown efficiency of TRE17 in HT1080 cells. *A*, HT-1080 cells were transfected with GFP-TRE17 and siCtrl #1 or siTRE17 #1. Expression of GFP-TRE17 was analyzed by Western blotting. *B* and *C,* HT1080 cells were transfected with siCtrl #1 or siTRE17 #1 and expression of endogenous TRE17 (arrow) was analyzed by Western blotting. Band intensities were quantified and presented as the fold change compared to the control. *D-F*, knockdown of TRE17 by another set of siRNAs, siCtrl #2 and siTRE17 #2, was analyzed as in *A*, *B*, and *C*. Graphs shown are mean ± SD from three independent experiments. ***p* < 0.01, Student's *t*-test.

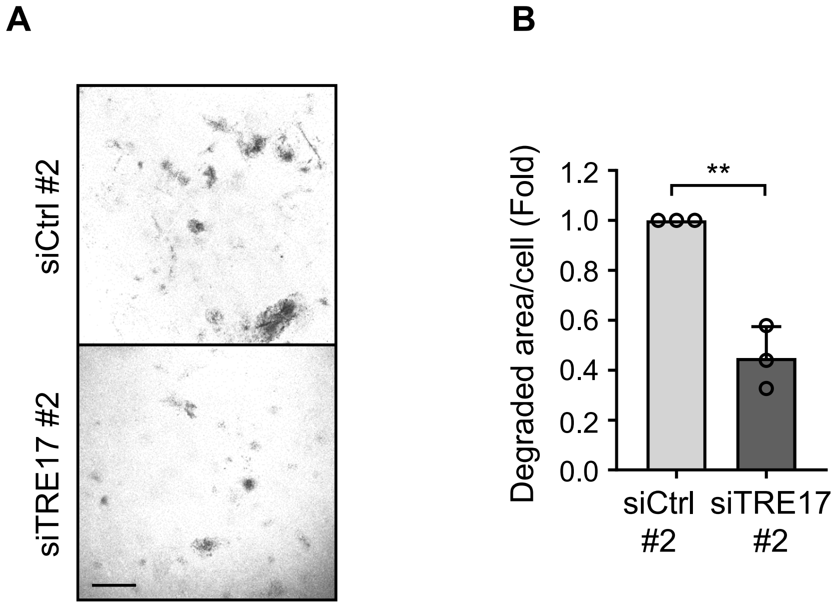

Figure S2. Knockdown of TRE17 attenuates the matrix degradative activity of HT-1080 cells. *A* and *B*, HT-1080 cells were transfected with siCtrl #2 or siTRE17 #2 and subjected to the gelatin degradation assay as in Figure 1, *C* and *D*. Scale bar, 100 μm. The graph shown is mean ± SD from three independent experiments. $^{**}p < 0.005$, Student's *t*-test.

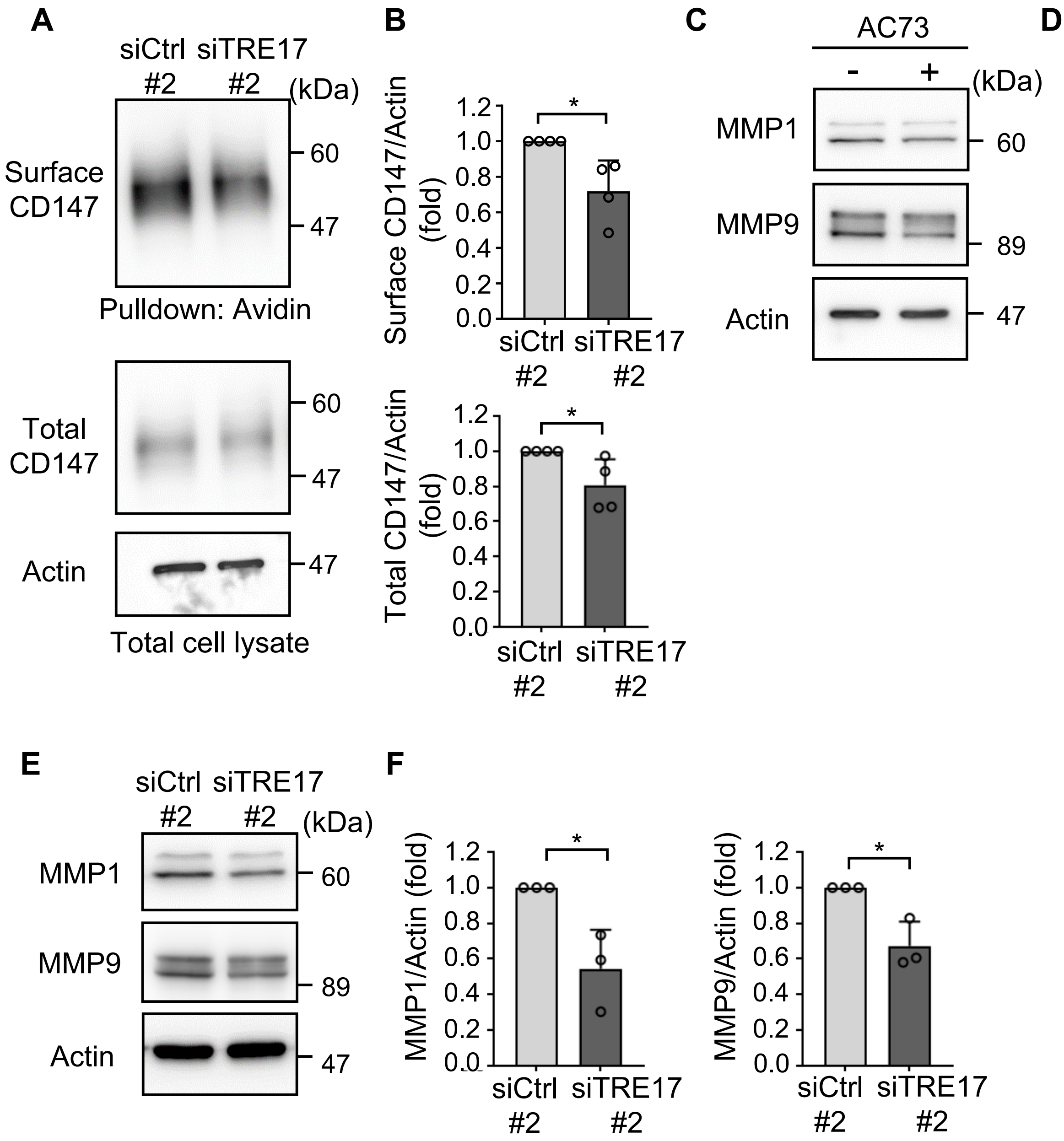

Figure S3. Knockdown of TRE17 decreases cell surface CD147 and inhibits MMP production. *A* and *B,* HT-1080 cells were transfected with siCtrl #2 or siTRE17 #2. Cell surface CD147 levels were analyzed as in Figure 2, *C* and *D*. *C* and *D*, cells were treated with or without 20 μM AC-73 and expression levels of MMP1 and MMP9 were analyzed as in Figure 2, *E* and *F*. *E* and *F*, HT-1080 cells were transfected with siCtrl #2 or siTRE17 #2 and expression levels of MMP1 and MMP9 were analyzed as in Figure 2, *E* and *F*. All the graphs shown are mean ± SD from at least three independent experiments. **p* < 0.05, **p < 0.005, Student's *t*-test.

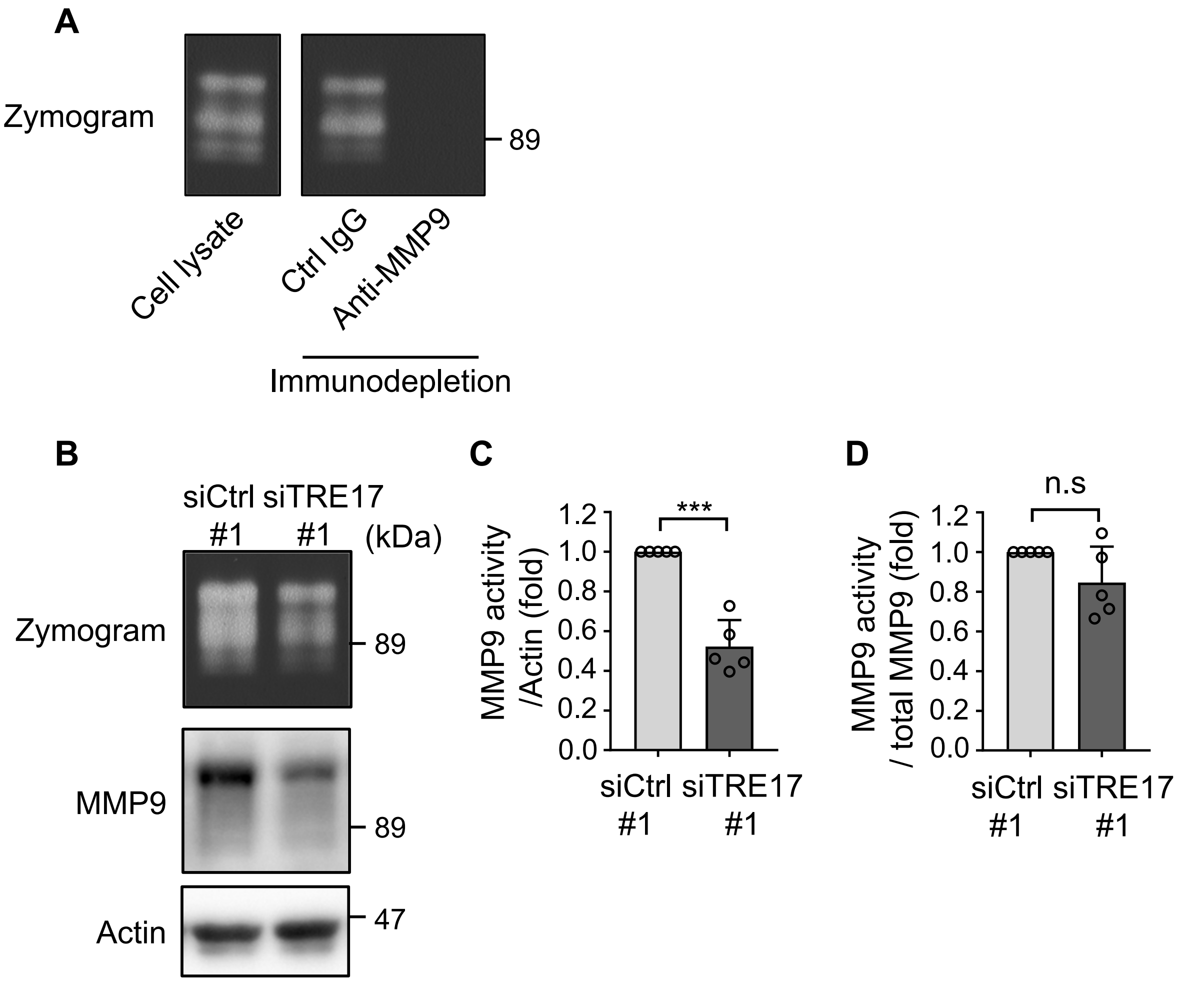

Figure S4. Decreased expression of MMP9 by TRE17-knockdown suppresses the gelatin degradative activity of HT-1080 cells. *A*, HT-1080 cell lysate was incubated with the anti-MMP9 antibody or control IgG and Protein G agarose. The control and MMP9-immunodepleted cell lysates were subjected to the zymography analysis. *B*, HT-1080 cells were transfected with siCtrl #1 or siTRE17 #2. The cell lysates were run on an 8% acrylamide SDS-PAGE gel with or without 1 mg/ml gelatin and subjected to a zymography analysis or Western blotting, respectively. *C* and *D,* band intensities of B were quantified. The values of the zymogram bands corresponding to the MMP9 gelatinolytic activity were normalized with those of actin blotting (*C*) and MMP9 blotting (*D*) and presented as the fold change compared to the control. All the graphs shown are mean ± SD from five independent experiments. ***$p < 0.001$; n. s., not significant, Student's *t*-test.

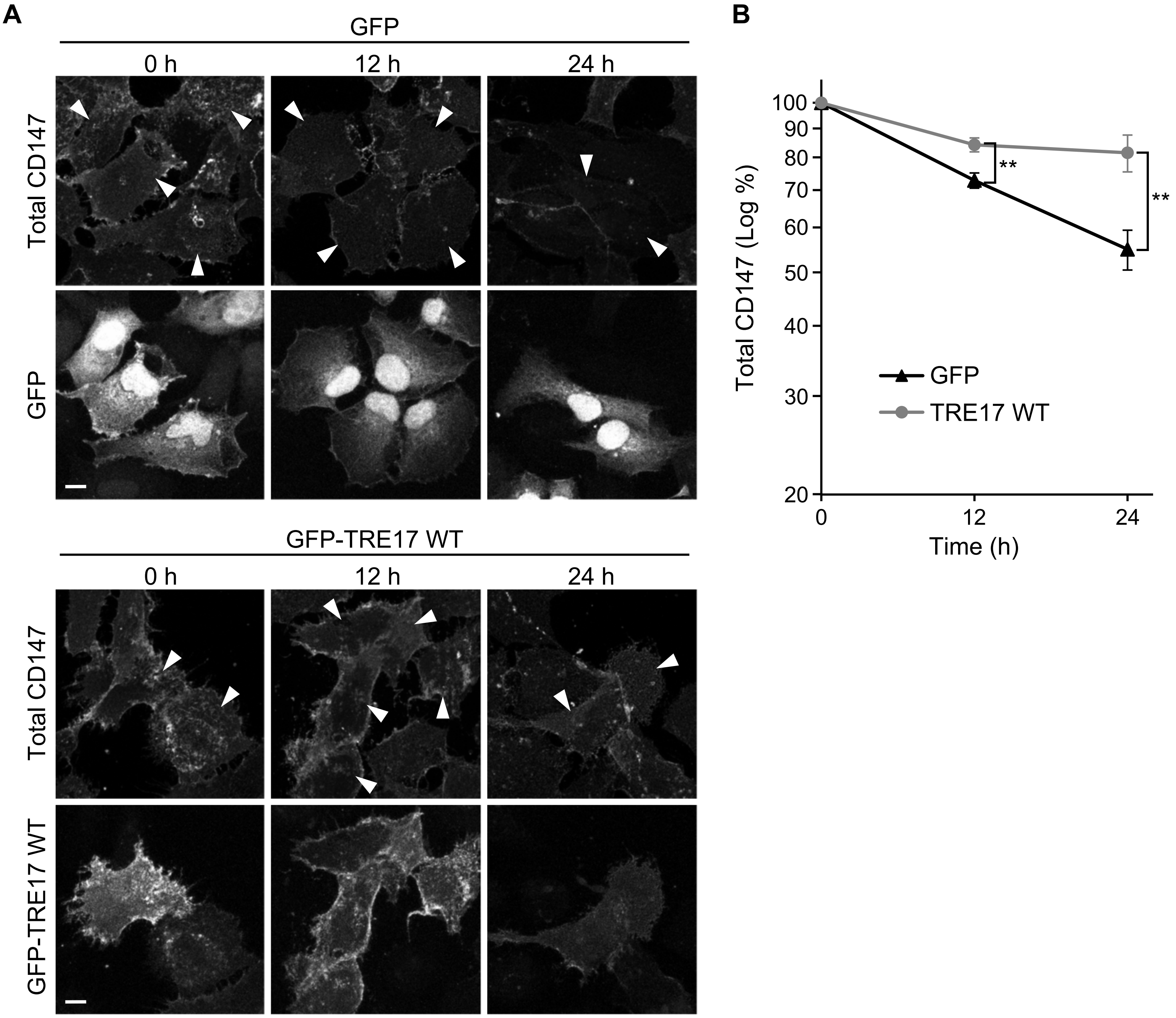

Figure S5. TRE17 delays degradation of CD147. *A*, HT-1080 cells transfected with GFP or GFP-TRE17 were subjected to the pulse-chase analysis as in Figure 5 except for the immunofluorescence staining with cell permeabilization to detect total pulse-labeled CD147. Arrowheads indicate cells transfected with GFP-tagged proteins. Scale bar, 10 μm. *B*, fluorescence intensities of total CD147 in *A* were measured and plotted as the percentage of the 0 h time point. Shown are mean ± SD from three independent experiments. $^{**}p < 0.005$, Student's *t*-test.
